# A Blind Spot? Screening for Mild Intellectual Disability and Borderline Intellectual Functioning in Admitted Psychiatric Patients: Prevalence and Associations with Coercive Measures

**DOI:** 10.1371/journal.pone.0168847

**Published:** 2017-02-02

**Authors:** Jeanet Grietje Nieuwenhuis, Eric Onno Noorthoorn, Henk Llewellyn Inge Nijman, Paul Naarding, Cornelis Lambert Mulder

**Affiliations:** 1 VGGNet, GGNet Mental Health Centre, Warnsveld, The Netherlands; 2 Dutch information Centre of Coercive Measures, Bilthoven, The Netherlands; 3 Resident training for psychiatrists, GGNet Mental Health Centre, Apeldoorn, The Netherlands; 4 Altrecht Aventurijn, Den Dolder, The Netherlands; 5 Epidemiological and Social Psychiatric Research institute, Erasmus MC, Rotterdam, Prins Constantijnweg 48–54, TA Rotterdam, The Netherlands; 6 Parnassia Psychiatric Institute, Rotterdam, Prins Constantijnweg 48–54, TA Rotterdam, The Netherlands; Maastricht University, NETHERLANDS

## Abstract

**Background:**

Failure to detect psychiatric patients’ intellectual disabilities may lead to inappropriate treatment and greater use of coercive measures.

**Aims:**

In this prospective dynamic cohort study we screened for intellectual disabilities in patients admitted to psychiatric wards, and investigated the use of coercive measures with these patients.

**Methods:**

We used the Screener for Intelligence and Learning disabilities (SCIL) to screen patients admitted to two acute psychiatric wards, and assessed patient characteristics and coercive measures during their stay and over the last 5 years.

**Results:**

Results on the SCIL suggested that 43.8% of the sample had Mild Intellectual Disability or Borderline Intellectual Functioning (MID/BIF). During their current stay and earlier stays in the previous 5 years, these patients had an increased risk of involuntary admission (OR 2.71; SD 1.28–5.70) and coercive measures (OR 3.95, SD 1.47–10.54).

**Conclusions:**

This study suggests that functioning on the level of MID/BIF is very prevalent in admitted psychiatric patients and requires specific attention from mental health care staff.

## Introduction

Many individuals with Mild Intellectual Disability and Borderline Intellectual Functioning (MID/BIF; IQ between 50 and 84) have difficulties in society and may also have problems with adaptive behavior [1,2]. Mild Intellectual Disability (MID; IQ between 50 and 70) is generally detected early in life, unlike Borderline Intellectual Functioning (BIF; IQ between 70–85), which is more often unknown to an individual, his/her family, or others[3]. Thirty-one percent of adolescents with BIF have been estimated to have poor social functioning, as well as other problems[4].

According to the definitions and methods used, estimates of the prevalence of MID/BIF in the general population vary greatly. In Western countries, the population prevalence of Mild Intellectual Disabilities (MID) is estimated to be 0.7–1.3% [5, 6]. On the basis of the normal distribution of intelligence in the general population, 2.14% would have a IQ in the 50–70 range (MID) and 13.59% in the 71–84 range (BIF).

The prevalence of MID/BIF does not seem to have been studied extensively in psychiatric adult patients, with the exception of some studies in MID patients [7, 8] or BIF patients [9], it is thus unknown in people with severe mental illness treated in inpatient and outpatient settings, and often seems to go unrecognized [9, 10]. Such unawareness is likely to result in inadequate treatment, more lengthy hospital stays, more use of coercive measures, and poor outcome [8, 11]. Due to patients’ lack of ability to verbalize their feelings and emotions, the difficulties they experience may be expressed more often in acting-out behavior and somatic complaints. These can be wrongly interpreted, leading to false diagnosis and treatment [8]. There are various special considerations which have to be taken into account in assessing and classifying psychiatric disorders in adults with MID, including among others associations between comorbid conditions and a patient’s communicative limitations; a patient’s impaired capacity for providing consent; an assessor’s response style; and an assessor’s use of information from multiple sources [12].

Although it is not known why MID/BIF is poorly recognized, it is probably due to problems in communication, such as verbal handicaps and a poor vocabulary. It may also be due partly to “streetwise” presentation on the part of various individuals, or to socially acceptable answers that conceal intellectual shortcomings[1]. The clinical presentation of symptoms in patients with MID/BIF may also differ from that in patients with a normal IQ.

If clinicians knew the prevalence of MID/BIF in everyday psychiatric practice, they might be aware of the need to diagnose it and to take account of any intellectual disabilities in their patients. However, validated tests for assessing IQ are time consuming—a problem that led to the development of the Screener for Intelligence and Learning disabilities (SCIL), a short 14-item questionnaire that provides global insight into a patient’s cognitive abilities and assesses the risk for MID/BIF.

In this study we therefore determined the percentage of patients suspected of having a MID/BIF according to the SCIL in patients admitted to two wards for acute psychiatry in two different general hospitals. Second, we checked whether MID/BIF was documented in medical charts. Third, we compared the demographic and psychiatric characteristics of the SCIL-positive and the SCIL-negative patients. Finally, we investigated the number of involuntary admissions and coercive measures in the SCIL-positive and the SCIL-negative patients.

## Methods

### Design and setting

This prospective dynamic cohort study involved patients admitted to two acute psychiatric wards in general hospitals located in a catchment area with 300,000 inhabitants in the eastern Netherlands. The study was conducted and reported in accordance with the STROBE guidelines for reporting observational studies[13].

### MID/BIF screening using the SCIL

A test consisting of 14 questions and minor tasks, the SCIL is intended to provide global insight into a patient’s cognitive abilities. It was developed specifically to detect MID/BIF (IQ 50–84) in people in various social-service and health-care settings, and in jails, police stations, and homeless settings. It was validated in an adult sample consisting of 318 participants from social workplaces, probation services, organizations that provide support to clients with (intellectual) disabilities living in the community, and also treatment facilities for addiction care and mental health problems, by comparing the scores on the SCIL with test results obtained on the WAIS III [14–16]. The reliability of the SCIL expressed in Cronbach’s alpha was good (0.83 in the sample of 318 adult subjects). The AUC- value was high (0.93 in the adult sample). With 19 or lower as cut-off score, the SCIL accurately classified 82% of people with MID/BIF. Similarly, about 9 out of 10 people without MID/BIF (89%) were correctly classified as having no MID/BIF [15, 16].

The SCIL identifies two categories: SCIL positive, i.e., patients with a high risk of having MID/BIF; and SCIL negative, i.e., those patients with a low risk. No specific professional degree is required to administer the SCIL. Before administering it, the nurses who participated in the current study received two hours of training, after which they first assessed 8 patients under supervision before performing assessments on their own.

### Patients

All patients admitted for more than 6 days between June 15, 2014 and June, 14 2015 were eligible for the study. The SCIL was administered by nurses who were not involved in treating these patients. The exclusion criteria were a patient’s lack of command of Dutch, or his or her lack of cooperation. To engage in the test, patients also had to be able to concentrate for at least 20 minutes, an ability that was determined by the nurses. If the patient showed acute psychotic or an otherwise severely disordered mental state, the SCIL was not administered until recovery allowed the patient to concentrate.

### Chart information

The following was extracted from digital medical charts: basic demographic data, such as age, gender, marital status, ethnic background, and psychiatric diagnosis (DSM-IV-TR, as assessed by the psychiatrist on the ward); and information on admission history, previous voluntary or involuntary admissions, and current or previous coercion. The medical charts were also read by a research assistant (a psychologist). For the latter there were three reasons: to confirm the information in question, to screen for any IQ data, and to screen the biography for any information on MID/BIF such as diplomas and broken school careers.

### Coercive measures

Coercive measures were rated prospectively during the current hospital stay by means of the Argus rating scale [17], a short instrument covering all coercive measures such as seclusion, restraint and enforced medication. The psychologist also read the medical charts to check the Argus figures for any involuntary admissions or coercive measures in the past five years.

### Analyses

As appropriate, chi-square statistics or a Student’s *t*-test were used to test the differences between three groups: not assessed with the SCIL, SCIL-positive, and SCIL-negative. Similarly, for all patients, SCIL positive or otherwise, odds ratios were calculated for 1.) patient characteristics, 2.) admission history, and 3.) having experienced coercive measures (as measured on the Argus rating scale [17]) during the current hospital stay or during hospital stays in the previous five years. The significance level for the analyses was set at an α of 0.05. The analyses were performed with SPSS version 20.

## Results

### Demographic and psychiatric characteristics

In the 12-month inclusion period, 314 patients were admitted for longer than 6 days, 208 of whom (66.2%) could be examined using the SCIL. In 106 patients it was not possible to administer the SCIL, mostly because patients were discharged before the assessment could be completed (N = 49), but also because they had an insufficient command of Dutch (n = 20), refused to participate (n = 6), or because very severe psychiatric symptoms were revealed during the test (n = 2). 29 patients were not assessed because staff had no time to gather data. Table 1 contrasts the patient characteristics of three groups of patients: those for whom it was not possible to obtain a score on the SCIL, those with a positive SCIL, and those with a negative SCIL.

**Table 1:** Comparisons between patients with no SCIL, SCIL-negative patients, and SCIL-positive patients.

	No SCIL	SCIL negative	SCIL positive	SCIL positive / SCIL negative		Difference between all groups	Difference between SCIL-positive and SCIL-negative patients
				OR	95% CI OR		
N =	106(33.8%)	117(56.3%)	91 (43.8%)				
Age	43.08	43.95	44.45			-	-
Male	49.1%	41.0%	37.4%				
Female	50.9%	59.0%	62.6%	1.16	0.66–2.04	-	-
**No partner**	64.2%	53.0%	68.1%			-	**+**
**Partner**	35.8%	47.0%	31.9%	0.53	0.30–0.93	-	+
**Western descent**	83.0%	96.6%	93.4%				
**Non-western descent**	11.3%	2.6%	6.6%	0.37	0.09–1.54	**++**	-
**Unknown**	5.7%	0.9%	0%				
Diagnosis							
No diagnosis*	4.8%	8.5%	12.1%	1.47	0.59–3.63	**++**	-
Anxiety disorder	5.8%	9.4%	8.8%	0.93	0.36–2.41	-	-
Depression	24.0%	44.4%	28.6%	0.50	0.28–0.89	**++**	**+**
Bipolar disorder	8.7%	8.5%	17.6%	2.28	0.98–5.34	-	-
Psychotic disorders	27.9%	16.2%	20.9%	1.36	0.67–2.75	-	-
Schizophrenia	11.5%	4.3%	4.4%	1.03	0.27–3.95	**+**	-
Drug-abuse disorder	17.3%	8.5%	7.7%	0.89	0.33–2.41	**+**	-
Personality disorder**	33.3%	26.5%	33.3%	1.36	0.75–2.48	-	-
Developmental disorder	11.4%	4.3%	14.3%	3.73	1.27–10.89	**-**	**+**
IQ data in charts	11.3%	11.8%	22.1%	2.12	0.98–4.57	**-**	**-**
Total IQ (score and n =)	86 (5)	89 (11)	69 (13)				
Performal IQ (n =)	83 (2)	101 (4)	86 (3)				
Verbal IQ (n =)	83 (2)	98 (4)	87 (4)				
WAIS (n =)	83 (4)	89 (9)	74 (11)				
Visual WAIS (n =)	83 (4)	87 (9)	70 (11)				
Working Memory Wais (n =)	83 (4)	93 (9)	64 (11)				
Processing speed Wais (n =)	83 (4)	81 (9)	62 (11)				

*: No diagnosis was found in the medical chart.

**: any personality disorder present.

OR = odds ratio— = no significant difference, + = significant difference, p<0.05, ++ = significant difference, p<0.01.

Ninety-one patients of the 208 patients who were screened with the SCIL (43.8%) were found to be SCIL positive. A higher number of patients in the SCIL-positive group than in the SCIL-negative group (OR 0.53 SD 0.30–0.93) had no partner. Fewer were diagnosed with depression (OR 0.50 SD 0.28–0.89). As may be expected, developmental disorders were more common in the SCIL-positive patients(OR 3.73 SD 1.27–0.89).

After comparing patients with no SCIL, the non-responders with those who were SCIL positive and those who were SCIL negative, the responders, we found that non-responders contained significantly more patients in three groups: those of non-Western descent (chi-square = 7.84, p = 0.02), those with drug-abuse disorder (chi-square = 5.56, p = 0.02), and those with schizophrenia (chi-square = 5.51, p = 0.02). It is likely that the options for assessing these patients had been impaired by language and attention problems.

The medical charts showed earlier documentation of an intellectual impairment in only a minority of the 91 SCIL-positive patients (22.1%). Even though IQ was documented in only a small number of patients, the mean IQ in the SCIL-positive group (n = 13) was 69, compared to 89 (n = 11) in the SCIL-negative group.

#### Coercive measures

With respect to coercive experiences, SCIL-positive patients had a higher risk of being admitted involuntarily (OR 2.71; SD 1.28–5.70, p<0.05). Their medical charts also reported a higher number of past involuntary admissions (OR 2.20, SD 1.12–4.32, p<0.01), and showed that patients who tested SCIL positive had had a higher risk of undergoing coercive measures (i.e., seclusion, restraint and forced medication) (OR 3.95, SD 1.47–10.54, p<0.01). Table 2 presents the outcomes for a number of coercion-related items in all SCIL groups.

**Table 2:** Use of coercive measures in patients with no SCIL, in SCIL-negative patients, and in SCIL-positive patients.

	No SCIL	SCIL negative	SCIL positive	SCIL positive / SCIL negative		Difference between all groups	Difference between SCIL-positive and SCIL-negative patients
				OR	95% CI OR		
	106 (33.8%)	117 (56.3%)	91 (43.8%)				
Currently involuntary admitted	20.8%	11.1%	25.3%	2.71	1.28–5.70	**+**	**+**
Admitted involuntarily in last 5 years	35.8%	15.4%	28.6%	2.20	1.12–4.32	**++**	**+**
Coercion* during current stay	4.7%	3.4%	7.7%	2.35	0.67–8.30	-	-
Coercion* in the last 5 years	19.8%	5.1%	17.6%	3.95	1.47–10.54	**++**	**++**
Ever admitted involuntarily	40.6%	23.1%	37.4%	1.99	1.08–3.63	**+**	**+**
Ever experienced coercion*	22.6%	7.7%	23.1%	3.60	1.56–8.31	**++**	**++**

*: coercion = seclusion, restraint and forced medication.

OR = odds ratio— = no significant difference, + = significant difference, p<0.05, ++ = significant difference, p<0.01.

## Discussion

Approximately 40% of recently admitted psychiatric patients were at risk for having MID/BIF as assessed on the SCIL. Only in 22.1% of the SCIL-positive group did the medical charts show an earlier diagnosis of MID/BIF. A SCIL-positive patient was more likely than a SCIL-negative one not to have a partner, and not to have been diagnosed with depression. SCIL-positive patients were more likely to have been admitted involuntarily and to have been subjected to coercive measures in the past.

If, as we assume, SCIL-positive patients are at a high risk of having MID/BIF, the prevalence of MID/BIF is much higher in the current study than in the general population. The prevalence of MID/BIF in our patients was comparable to that in an unpublished study on patients treated in Assertive Community Treatment teams, which found a prevalence of MID/BIF as high as 59%. The study in question had assessed MID/BIF using a Dutch Intelligence Test (GIT) [18]. Another study in ACT teams in Ontario, Canada, estimated MID to be 9–11% [19]. We found no other studies that had assessed both MID and BIF in psychiatric patients.

Our finding that fewer SCIL-positive patients than SCIL-negative patients had a depressive disorder contrasts with a study showing that affective disorder is one of the commonest disorders in people with MID [8]. As we also know from the Diagnostic Manual—Intellectual Disability (DM-ID) [20] there may be differences between the clinical presentation of symptoms in patients without intellectual problems and the presentation in the patients with MID (and often BIF) we know in clinical practice. When depressed, adults with MID have been noted to have higher rates of conduct problems, social withdrawal and irritable mood [8]. In the same study, developmental disorder was diagnosed in 11.4% of the SCIL-positive group, against 4.5% in the negative group. These findings are confirmed in studies by Prasher et al [21] in which an Autism Spectrum Disorder was associated with MID. In our SCIL-positive sample, we note the modest number of patients diagnosed with substance-use disorder (SUD). A study across several samples by Duijvenbode et al [22] showed that the prevalence of SUD with MID/BIF varied very widely (0.5–21% or more).

Our results also show that, in the past, SCIL-positive patients had had more involuntary admissions than SCIL-negative ones, and had experienced more coercive measures. This is a remarkable finding. Coercive measures may obstruct recovery and even result in iatrogenic PTSD [23]. People with MID/BIF have reduced coping skills, and easily react with verbal aggression, and, in circumstances they cannot oversee, with abject behavior or refusal behavior. In the context of an admission ward with large numbers of severely disordered patients, their inability to cope may even increase, impairing diagnosis and the identification of treatment that will meet their needs. To prevent coercion, MID/BIF patients treatment should thus be adapted to their intellectual capacities and to their ability to understand their environment.

### Strengths and limitations

#### Strengths

To our knowledge, this is the first prevalence study on MID/BIF in a relatively large sample of acutely admitted psychiatric patients. Even though the assessments were performed on acute psychiatric wards, we were able to include a substantial number of patients (66.2%). In such a setting, this is a very reasonable response rate[24]. As the data were gathered in two psychiatric wards in general hospitals, our findings have validity for clinical practice. Because there were no specific selection criteria for admission to these wards, these findings may be a good overall reflection of psychiatric patients admitted in the Netherlands.

#### Limitations

The first limitation is that the SCIL is a screener instrument for assessing MID/BIF that was not followed by a fully validated IQ test, such as the WAIS, and then—as might be preferred—by a second test for emotional and adaptive functioning.

The second limitation is that we do not know what causes a SCIL-positive result: it may result from cognitive decline, from psychiatric disease or symptoms, or from long-term psychiatric medication. Nevertheless, whatever the origin of the intellectual impairment, our study shows that a high proportion of patients who are admitted in the acute phase of psychiatric disease appear to function at the level of MID/BIF, and that their treatment and handling should be adapted to their specific abilities and care needs. To what extent the intellectual impairment with which they present is an inborn defect or the effect of cognitive decline or is a subject for further research.

A third possible limitation is that there may have been a selection bias involving patients with schizophrenia, drug abuse and those of non-western descent.

Finally, no structured interview was used to establish the DSM-IV–TR diagnosis.

#### Recommendations for clinical practice and research

To assess their earlier intellectual aptitude, we recommend that all patients admitted are interviewed with regard to their school career, diplomas, and employment. To understand the impairments of a patient with MID/BIF, clinicians have to adapt their communication and attitude. This can help to avoid coercion, and can also support the recovery process.

Although the SCIL is not a cognitive test in the narrower sense, and although many skills required to perform the small tasks in the SCIL are learned in early life, we do not yet know the influence of psychiatric symptoms on the SCIL test result. We therefore recommend further research on the association between SCIL findings, psychiatric symptoms, medication, and drug use or drug abuse that possibly results in cognitive decline. As well as examining intellectual capacities with an intelligence test, one might consider other assessments of adaptive functioning and social emotional development. Finally, we recommend that the current study is repeated in first referral patients, Functional Assertive Community Treatment (FACT) patients, and long-stay patients.

## Conclusions

Overall, we feel it is fair to assume on the basis of our results that MID/BIF is present in a substantial proportion of admitted psychiatric patients, and that such a diagnosis may often be overlooked in the acute phase of psychiatric disease. Findings show a relatively high number of patients with a suspected MID/BIF, next to a substantial lack of information on education and employment history in their biographies. To assess their earlier intellectual aptitude, we recommend that all patients referred to mental health treatment are interviewed with regard to their school career, diplomas, and employment. To understand the impairments of a patient with MID/BIF, clinicians have to adapt their communication and attitude. This may provide an impression of formal intellectual functioning, can help to avoid coercion, and can also support the recovery process.

It is also important to gain an impression of a patient’s intellectual capacities—by using the SCIL, for example—and of their social function skills and adaptive behavior. Examination of the patient’s intellectual capacities through an intelligence test might also be considered. Failure to identify an MID/BIF may represent an additional risk with regard not only to involuntary admission, but also the use of coercive measures that may lead to iatrogenic damage. The need to identify this particular patient group at an earlier point in treatment is an important challenge in mental healthcare.

## Supporting Information

S1 Data The file scildataplosone.zip contains the underlying data for data sharing purposes. The first line of the database formatted in Excel contains the variable labels. Please contact the second author on e.noorthoorn@ggnet.nl when data are pooled.
(ZIP)
